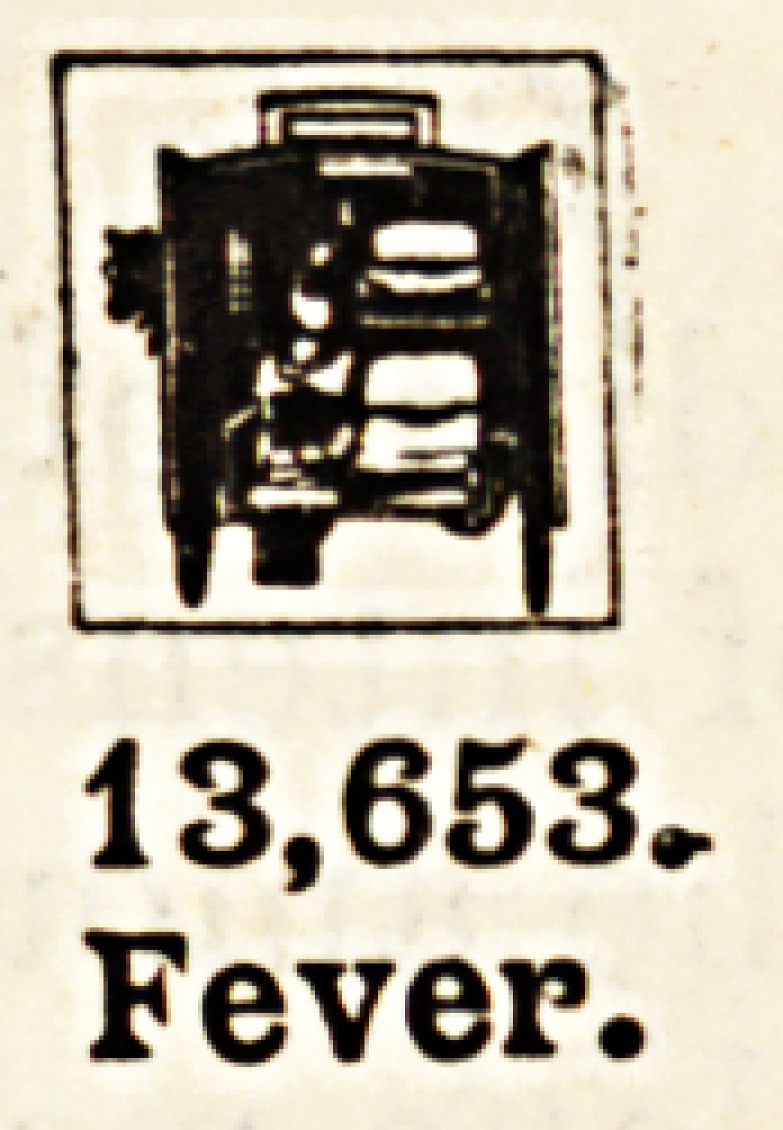# Special Hospital Sunday Supplement

**Published:** 1902-06-14

**Authors:** 


George P.

Photo by W. & D. Downey, Ebury Street, London.

Victoria Mary

1902

Photo by W. & D. Downey, Ebury Street, London.

### Special Hospital Sunday Supplement.

### "Our Portraits."

### THEIR ROYAL HIGHNESSES THE PRINCE AND PRINCESS OF WALES.

We are presenting to our readers with this issue two admirable photogravure portraits of their Royal Highnesses the Prince and Princess of Wales. Both the Prince and Princess are taking a most active part in helping in the work which the hospitals are doing. Their example is most valuable, and their sympathy has so much endeared them to the people that we feel our portraits will be both welcome and appropriate at the present time. They will probably find a place in the hospital boardrooms and nurses' rooms throughout the Empire.

### The King and the Sunday Fund.

The best motto for a king is surely "Stand for an ensign to the people." In the Coronation Year, when the King has expressed a wish that all those who desire to make a gift to himself should do so in money and place it to the credit of the Hospital Fund, it is essential that every effort should be made as loyal subjects and citizens of the metropolis of the Empire to combine together to make the Hospital Sunday collections in such a year a record for all time. The King has unfurled the ensign, and asks the people to rally to the standard. Preachers of all denominations have here given to them a text which should stir their hearts. Will they not make special efforts to secure that every one of their hearers on Hospital Sunday may be made to realise the beauty of personal service in the cause of the sick. Indeed, all who are loyal, nay all who are true men and women, should signify their loyalty and happiness at this time by a liberal contribution to the hospitals. It is good to be a king, but it is better to be a king beloved and trusted by his subjects. Who is more entitled to praise and honour on Hospital Sunday than a ruler like King Edward VII., who has declared that nothing appeals more deeply to himself than the cause of the hospitals? The King asks for the most liberal contributions to these splendid institutions in the year of his Coronation as a testimony of the loyal generosity evinced towards their King by the people.

In considering the attitude of his Majesty the King to the hospitals on the present occasion, especially in connection with the Hospital Sunday Fund, which is necessarily our text to-day, it is well to recall the fact that in his letter of February 5th, 1897, instituting the King's Hospital Fund, his Majesty used these words: "We shall not trench upon the ground occupied by the Hospita Sunday Fund; but for the first year we shall rely upon the co-operation of the distribution committee of that Fund." During the five years which have elapsed, the authorities of the two Hospital Funds have cordially co-operated together, and it is satisfactory to note that, whereas the highest amount received by the Hospital Sunday Fund in any year, with one exception when a supreme effort was made, up to and including the year 1896, was £46,035, the average total receipts for each of the five years, 1897 to 1901, since the King's Fund was instituted, has been £48,326. We have here evidence not only that the King's express object has been fulfilled, but that competition in good works has as usual tended to strengthen the older organisation. This view is confirmed by the circumstance that, whereas the contributing congregations in the year 1897 amounted to 1,793, they had increased to 1,820 in 1901. It is, however, matter for regret that the appeals from the pulpit, whether they have failed in urgency or not, have failed to be as successful during these last five years, as they were formerly. In other words, the average contribution per congregation is smaller than it used to be in the early days of the Hospital Sunday Fund organisation in London. Can it be that the influence of the Churches is diminishing, or is it the zeal of the preachers in the cause of the sick? It is clearly not due to lessened liberality on the part of the laity independent of the Churches, for the receipts from legacies, special donations, and dividends have steadily increased during the last five years, having risen from £3,607 in 1897 to £18,343 in 1901, a circumstance which shows the confidence which the general public has in the method of distribution adopted by the Council of the Sunday Fund.

### The Prince of Wales and Personal Service.

There is no training like that which a man receives in the British Navy. For fertility of resource, whole-hearted devotion to duty, intelligent appreciation of plain facts, and a readiness to render aid to the fullest extent of a man's powers, the British sailor properly stands without a peer in the judgment of his countrymen. The Prince of Wales possesses to a high degree all those qualities which make the sailor so popular a personage with the British people. His Royal Highness does nothing by halves, and since he entered upon the position of Prince of Wales after his return from that memorable voyage round the Empire, he has shown to a remarkable degree the strength of his virtues in the discharge of his public duties. Accompanied by that charming personality, the Princess of Wales, His Royal Highness has visited systematically the various metropolitan hospitals which have a special claim upon his consideration, and the manner of his visit and the method of his inspections have surprised and pleased everybody who is acquainted with the facts. It has been claimed over and over again that the voluntary system of hospital support is vital to the well-being of British hospitals. If that claim is to be justified, our hospitals must be capable of standing the criticism and close investigation of all who know most about their administration. This view is evidently held by the Prince of Wales and may account in a measure for his thoroughness as a hospital visitor.

When visiting a hospital the Prince makes a point of providing that no preparations shall be made for his visit, and that the time of it shall not be known to anyone until the day it takes place, and then only to the chief official whose duty it will be to conduct him over the institution. Those who have had most experience in the work of inspecting hospitals will agree that if a sound judgment of the efficiency of their management is to be formed, it is essential that the inspector should take an opportunity of visiting all parts of the establishment without previous notice. In this one fact, the manner of the visits paid by the Prince of Wales to the principal London hospitals during the last few months, we have convincing proof of the thoroughness and interest which he takes in the work, and the knowledge which he brings to bear upon his investigations.

Of course, the first thing to be borne in mind is that a hospital contains a number of individual sufferers, and that each of them has an interest all his own. As the well-being of the patients is the first consideration to be thought of, so the hospital visitor first directs his attention to the patients. Their Royal Highnesses, on entering a ward, arrange to go to the bedside alone, that they may converse uninterruptedly with the patients, and may make any inquiries of them which they may wish to put. The evident interest and quick sympathy invariably displayed by the Prince and Princess are naturally much appreciated by the patients. But what has most impressed all who have been present when the Prince has inspected a hospital is his evident determination to treat the visit as a private function, to stick closely to business, to avail himself of every opportunity to make himself more fully conversant with the details of the working of hospitals in London, and to leave nothing undone that may assist him in attaining the great object of the King and himself—to free the hospitals from debt, and secure the opening of all unoccupied beds.

The practical methods of the Prince are indicated by his examination of the design of each hospital, and the plan upon which it is built. He criticises the appearance of the wards, their size, the kind and quality of the bedsteads, flooring and fittings; makes close inquiries as to the methods of heating and ventilation; visits the kitchen; inquires as to the composition of the kitchen staff, the economy practised, the number of meals served, and the method of their distribution. The Prince has evidently a good knowledge of machinery, and does not fail to note whether the arrangements of the lifts and food trolleys are such as to economise labour to the fullest extent. Everywhere he endeavours to stimulate the hospital authorities to introduce the best appliances, and where, as in some cases, straitened finances have prevented the authorities from introducing the electric light, he has not hesitated to enforce his opinion that such an improvement is nowadays practically a necessity. If, as is the case in some of the older hospitals, refurnishing has

### WHAT EACH INSTITUTION SPECIALLY NEEDS.

**Alexandra Hospital.**—Two wards waiting to be opened.

**Belgrave Hospital.**—Grosvenor ward closed; hospital severely taxed in consequence.

**Birmingham General Hospital.**—Deficit for 1901 over £5,000 (see p. 27).

**Bolingbroke Hospital.**—New buildings being erected; donations wanted to open free of debt.

**Bournemouth Sanatorium for Consumption.**—Deficit for 1901, £432; £2,900 has to be raised annually from voluntary sources (see p. 26).

**British Lying-in Hospital.**—Another ward in contemplation.

**Cancer Hospital, Brompton.**—Reliable income short of expenditure by £6,000 (see p. 6).

**Central London Ophthalmic Hospital.**—Rebuilding cannot be commenced until £5,000 has been obtained (only £3,000 at present).

**Charing Cross Hospital.**—Rebuilding taking place £25,000 required to complete first part.

### THE PRINCE OF WALES AND PERSONAL SERVICE.—*Continued.*

become necessary, the Prince and Princess have invariably notified the fact, and in more than one instance, with a view to encourage the authorities to increase the average of efficiency by refurnishing, they have offered to give new bedsteads for an entire ward. Further, in certain hospitals, the Prince has been apt to notice marked efficiency, and before leaving has intimated his intention to pay a second visit so that he may complete the inspection of every department and learn everything which the high efficiency of the methods pursued can teach him for the purposes of his inspection work.

But there is another side to the Prince's influence for good in connection with our hospitals. On a recent occasion he made the astounding statement that privately by his own efforts he has succeeded in obtaining promises which will yield a revenue of £20,000 a year at least to the metropolitan hospitals. May we not conclude that this magnificent result is in a measure the working of cause and effect? The visits of inspection have quickened the interest and familiarised the Prince with the needs of our hospitals. The Prince then, practical and good man that he is, sets to work with determination and secures an additional £20,000 a year. Here surely is a fact which preachers and writers in the press who may co-operate to make Hospital Sunday in London a success, may drive home with advantage. We have held, as the late Sir Andrew Clarke held, that if the clergy, or the churchwardens, or the laymen, or the ladies, some or all of them in each congregation, would give a little personal service by organising a Hospital Society within the congregation, and then fix a hospital day in each year when such members of the church as were interested could visit a hospital, the results must be beneficial to the institution and individuals too. There are very many kinds of hospitals; each type has its own special methods and points of interest, and there is no secretary of any hospital in London who would not gladly make arrangements to receive the visitors from any contributing congregation to the Hospital Sunday Fund, and to make their visit to his institution in every way successful. We want more personal service, a wider spread of the spirit of individual earnestness of purpose, a keener individual recognition of the privilege of taking each his part in providing for the wayfarer who from no fault of his own falls out by the wayside in the battle of life. We do not urge this view merely in the interests of the hospitals, but because we believe that every man and every woman who will follow the example set by the Prince of Wales in this matter will find, as all who have tried it have found, that their own individual happiness will thereby be greatly increased. The sick and the suffering we have always with us, but how little do the majority remember this circumstance or recognise the duty and privilege of giving a little of their time, during the days of health and prosperity, to minister to the needs of the least fortunate of their neighbours.

### THE VICTORIES OF MODERN MEDICINE.

If we consider human suffering in the mass and think how it would be, in the presence of it all, to have to stand by helpless—deprived of all the aids which modern science gives for its alleviation—and if we further consider that all these means of alleviation come originally from the hospitals, we begin to grasp the untold benefits which these institutions have showered upon mankind, quite outside and beyond what they have done for that far more limited number who have come as patients within their walls. We will not here labour the matter or do more than mention the enormous diminution of human suffering which has arisen from the discovery of anæsthetics, or from the still greater discovery that many diseases are due to the growth of micro-organisms, and that by preventing their growth, as for example by antiseptics, these diseases may be prevented.

All this has now become a thrice told tale. But we may point out how immensely these discoveries, besides relieving the sufferings of those who of necessity had to come under the surgeon's knife, have increased the range of the surgery, bringing the possibility of relief to a vast number of cases which in times gone by no surgeon would have dreamed of touching. If we look back at the time when in all except the more trivial forms a compound fracture, especially if it involved a joint, meant amputation or death; when for a woman to suffer from an ovarian tumour meant that she must be tapped, and tapped, and tapped, until she died exhausted; when abdominal operations were desperate ventures; when operations on the brain were undreamed of; when surgeons occupied themselves in inventing wondrous means of operating without the knife—by cauteries, by écraseurs, by caustics, by anything to avoid the infliction of a raw wound—so great was their dread of that terrible blood-poisoning by which so large a proportion of their patients were carried off; and when we think of the work of Lister and those who have followed him, always labouring in hospitals, work which has resulted in the surgery of to-day, which extends its

### WHAT EACH INSTITUTION SPECIALLY NEEDS.

**Chelsea Hospital for Women.**—No endowment or reserve funds (see p. 3).

**City Orthopædic Hospital.**—Further alterations and a debt of £10,000.

**Convalescent Home for Poor Children, St. Leonards-on-Sea.**—£450 required to supply annual deficiency (see p. 31).

**Dental Hospital.**—Building and site mortgaged for £55,000.

**East London Hospital for Children.**—Extensions in contemplation to cost £20,000 (see p. 2).

**Great Northern Central Hospital.**—£8,000 to be raised from voluntary sources (see p. 23).

**Guy's Hospital.**—Asking for £180,000 and increase in voluntary support (see p. 8).

**Hampstead Hospital.**—New Hospital. £15,000 wanted.

**Hospital for Diseases of the Skin.**—New building wanted for in-patients.

**Hospital for Epilepsy and Paralysis.**—New hospital being built. £8,000 required for first section.

**Hospital and Home for Incurable Children, Maida Vale.**—Deficit for 1901, £300 (see p. 29).

### THE VICTORIES OF MODERN MEDICINE.—*Continued.*

life-saving efforts into every corner of the frame and into almost every disease in the nosology, we begin to grasp something of the indebtedness of mankind to the hospitals which have made such discoveries possible. We do not grasp it thoroughly, however, until we remember that these modern methods are now being put in operation not in hospitals alone, but in every quarter of the globe where sickness and suffering exist, and that what has been discovered and taught in hospitals is being put in practice in country villages, in mining towns, in camps, in missionary stations all over the world.

The great conception of the microbic origin of disease, a conception which originated in the hospitals, and the applicability of which to explain the various diseases to which flesh is heir is now being tested and examined in every hospital in the country, is also at the root of that great branch of the medical art which we speak of as preventive medicine, a department which, so far as life-saving is concerned, is likely to prove of the greatest public advantage. The greater part of the science of public health and of all that springs from it, the constant warfare against typhoid and diphtheria, the notification and removal of infectious diseases, the inspections and disinfections which are now provided by public bodies as a recognised part of municipal management, the clearing of unhealthy areas, the enforcement of measures for preventing the pollution of rivers, and for the provision of pure water, and, indeed, the whole fabric of that public health legislation which has produced so great a fall in the death-rate during the last quarter of a century, are but the practical application of doctrines which have sprung directly from hospital work.

To those who have time to investigate the statistics of the hospitals, one of the most interesting points which a comparison of recent with former years brings out is the much shorter time the patients now stay in hospital than they used to do. To some extent, perhaps, this is the result of the growing pressure upon their space and the increasing urgency of the demands for admission to their wards. The fact that a bed in one of our great hospitals is the safest of all safe places for anyone who is ill has been driven home among the working classes in London by personal experience. The people who know best, those who have again and again been in the hospitals themselves, are found in an ever-increasing crowd bringing up their sick to be cured, and clamouring for admission. Thus there is pressure upon the wards, and thus to some extent may we perhaps account for the shortened stay in hospitals. But certainly there is another factor in operation, and that is the far quicker recovery of serious cases under modern treatment. This again is a matter which it is impossible to bring to the test of actual figures, so different are the conditions now to what they were 30 years ago. But no one can watch what goes on in hospitals nowadays, or consider the long list of operations done in some of them, without seeing that to get through the work at the old speed would be quite impossible. All of this means not only a saving of suffering, but, what to the working man is of almost as great importance, namely, a saving of time. The saying "what thou doest do quickly" applies with double force when it concerns the cure of a working man who has a family dependent upon him, and there is no doubt that the modern hospital does "do quickly" what it undertakes, as is shown by the shortened stay in hospital which is now required for the cure of many diseases; all of which is of immense advantage to hospital patients. Let us, however, leave the individual patient and turn again to what hospitals and their work are doing for the world. Look at the great problem of malaria and its prevention, which is now being worked out in connection with the hospitals and more particularly with the London School of Tropical Medicine, a problem on the solving of which the extension of English colonisation so largely depends; look at what is being done to show how we may best wage war against tuberculosis; look at the Finsen light treatment, the use of X-rays for various diseases, and its startling success in certain forms of cancer; look at the poor consumptives actually being cured by open air and high feeding in our hospitals although they had previously been dying by inches under the combined influences of under feeding and impure air in their own homes; look at all the work which is going on, and we see that not only are the hospitals doing a great and almost indescribable charity to those who having had the evil fortune to get ill have had the good fortune to have been received by these noble institutions, but that they are doing something even greater for mankind at large, in that they are the workshops in which medical knowledge is patiently hammered into form and whence when finished it is continually poured forth freely and without restrictions for the use of the whole world.

Out of pure charity as a mere gift from man to man, we beg you to give to the hospitals. Out of pure philanthropy as a means of lessening the sum total of human suffering, we also beg of you to give to the hospitals which do so much to lighten the burden which man has to bear. But out of pure humanity we still further beg that you will give to hospitals which act as founts of knowledge by aid of which, not here alone but all over the wide world, life is lengthened and rendered more useful and more happy.

### WHAT EACH INSTITUTION SPECIALLY NEEDS.

**Hospital for Sick Children, Great Ormond Street.**—Requires £7,000 to make good annual deficiency (see back cover).

**Hospital for Women, Soho Square.**—An addition of £2,000 to its present income wanted to maintain its 64 beds (see p. 6).

**Irish Distressed Ladies Fund.**—Needs £630 to remove deficiency on last year's account. No reliable income (see p. 28).

**King's College Hospital.**—£75,000 urgently needed to meet present debt and liabilities and to carry out necessary improvements (see p. 6).

**London Fever Hospital**—Reliable income falls short of necessary expenditure by £3,000 (see p. 26).

**London Homœopathic Hospital.**—Annual deficit over £2,000 (see p. 25).

**London Hospital.**—Total outlay necessary not less than £370,000 (see p. 5).

**London Lock Hospital.**—In debt to bankers (see p. 24).

**Mary Wardell Convalescent Home for Scarlet Fever.**—£1,000 urgently wanted (see p. 31).

**Metropolitan Convalescent Institution.**—About £11,000 required annually for the four homes (see p. 31).

### A WORD TO LIVING LONDONERS.

### WAYS AND MEANS.

YEAR by year in our Special Hospital Sunday Supplements we have given statistics to show the different proportions of the money given for the care of the sick in London by the living, namely, the present inhabitants of the Metropolis, by deceased benefactors and by the patients themselves; and we have pointed out how inadequate is the sum contributed by the living to insure the good work which is done by our hospitals being maintained. The returns for 1900 are not calculated to make us particularly satisfied with ourselves. We find that in this year, including St. Bartholomew's Hospital, the voluntary contributions amounted to 8s. 7d. in the pound, which, added to the 1s. 7d. received from the patients themselves, bring the amount given by the living up to 10s. 2d. in the pound; an increase, it is true, on the amount received in 1899, but entirely due to the increased payments by the patients. When, therefore, Londoners are inclined to feel proud of their exceeding liberality to the hospitals they deceive themselves. That is a mean and contemptible spirit which takes credit for virtue it does not possess. If no such reproach is to rest on London this year it is imperative that all classes should give more liberally to the Hospital Sunday Fund and persuade others to follow their example.

### THE INCOME AVAILABLE FOR THE WORK DONE.

In the year 1900 hospital treatment was provided for nearly one million nine hundred thousand persons, exclusive of the patients treated at the hospitals of the Metropolitan Asylums Board, and the total income received by the London voluntary hospitals and dispensaries for this purpose was £991,661, which was derived from the following sources :—

| | | |
|---|---|---|
| Charitable or voluntary contributions ... ... | £428,741 | or 43 per cent. |
| Income from invested property | 277,069 | „ 28 „ |
| Legacies ... ... ... | 208,541 | „ 21 „ |
| Patients' payments ... ... | 77,310 | „ 8 „ |

So far as the above figures refer to St. Bartholomew's Hospital they have been confined to that portion of the revenue which was applicable to hospital purposes.

### HOW THE MONEY IS PROVIDED.

The mere statement that such and such an amount of money has been raised from one source or another does not, as a rule, greatly impress people. It is rather the difficulties which attend the raising of it, and the consideration of the sources from which it comes, that enable us to judge whether those who are in a position to give are doing their duty. It is therefore of the first importance that everyone should thoroughly understand where the money comes from to pay the cost of the relief given by the hospitals to the inhabitants of London, and to make this as clear as possible we have prepared diagrams, each representing a hand and a coin, which have been drawn to scale to show the proportion of every sovereign contributed by the living, *i.e.*, those who receive the benefits, and by deceased benefactors, many of whom have not only left their money to enable the good work to be carried on, but were also during their lives active workers for the hospitals. With a view to clearness the diagrams have been drawn to represent the proportion of every sovereign

THE DEAD HAND GIVES 9S. 10D. OUT OF EVERY £1 RECEIVED BY THE HOSPITALS.

given in 1900 by (*a*) the dead, (*b*) the living, and (*c*) the patients themselves. *The black hand* and the coin held by it represent the contributions from those now dead; *the white hand* represents the charitable contributions from the living, *i.e.*, ourselves, the living Londoners; and the smallest coin indicates the amount received from patients' payments.

Of every sovereign received 9s. 10d., or nearly *one half, is derived from legacies and the interest on gifts* from deceased benefactors, which have been invested in approved securities; 8s. 7d. out of every sovereign has been *given in charity* by the present inhabitants of London, that is, *the living, for the benefit of whose generation the hospitals exist*; and 1s. 7d. of every sovereign has been contributed by those *who have been actually under treatment in the hospitals*. Let us study these figures carefully, and understand why they are so unsatisfactory, why they do not inspire us with confidence. It will be seen that a considerable portion of the 9s. 10d. given by the dead hand is derived from the legacies received

### WHAT EACH INSTITUTION SPECIALLY NEEDS.

**Metropolitan Hospital.**—Over £9,000 required annually, and £3,254 to meet deficit for 1901 (see p. 25).

**Mount Vernon Hospital for Consumption.**—Wants £15,000 to complete extensions, and £6,000 to make good deficiency in annual income (see front cover).

**National Hospital for Heart Disease, Soho Square.**—Requires £1,750 from voluntary sources for yearly maintenance (see p. 27).

**North Eastern Hospital for Children.**—Scheme for necessary enlargement in hand.

**Paddington Green Children's Hospital.**—£2,300 in debt (see p. 28).

**Poplar Hospital.**—Have built three new wards, but only two opened (see p. 24).

**Queen Charlotte's Lying-in Hospital.**—Annual deficit of £3,000 (see p. 3).

**Royal Ear Hospital.**—Awaiting funds to rebuild (see p. 29).

**Royal Free Hospital.**—£1,500 still required for structural improvements (see p. 26).

**Royal Hospital for Children and Women.**—To be enlarged and rebuilt.

**Royal Hospital for Diseases of the Chest.**—Annual deficit £5,000 (see p. 7).

### A WORD TO LIVING LONDONERS.—*Continued.*

during the year—to be exact, 4s. 3d. of it—and this source of income must always be fluctuating and unreliable. When dealing with these figures last year we laid particular stress upon this fact, and pointed out that because the amount received from legacies happened to have steadily increased during the previous two or three years that was no reason why we should believe this source of income to be a certain one. We gave the warning that what had

THE LIVING, *i.e.*, THE PRESENT INHABITANTS, GIVE 8S. 7D. OF EACH £1 RECEIVED BY THE HOSPITALS.

occurred in 1896, when the legacies showed a decrease of £40,000, might at any moment happen again. This year it has happened. The amount received from this source in 1900 was over £70,000 less than that received in 1899. Such a large and sudden falling off in revenue shows us how perilous it is to trust to the dead hand which may fail us, not only for one year, but for a succession of years. It brings us face to

PATIENTS' PAYMENTS SUPPLY 1S. 7D. OF EACH £1 RECEIVED BY THE VOLUNTARY HOSPITALS.

face with the fact that, unless such deficiency is made up by the increased gifts of the living, the great work of our London hospitals must be seriously crippled.

Now let us see how the gifts of the charitable compare this year with last. At the first glance it would appear that they were at least up to the standard, 8s. 7d. in the sovereign having been contributed in both years; but in reality this figure is only maintained because the total income of the hospitals was less. As a fact, the receipts from the charitable were £43,000 less in 1900 than in 1899, so that had the receipts from legacies been maintained, the charitable would have given only about 8s. in the sovereign, instead of 8s. 7d. It may be urged that as these figures refer to 1900, and that during that year the war in South Africa made special claims upon the people of this country, comparison should not be insisted upon; but is this not rather a flimsy excuse? We very much suspect that the claims of the war have been put forward by many who never gave a fraction to the war funds or to any other funds either. It is so easy to find excuses for shirking one's duty. Against this deficiency of the charitable it is pleasant to record that the amount received from the patients themselves shows a well-marked increase, from 1s. 3d. in the £1 to 1s. 7d.

### The Meaning of the Diagrams.

We most earnestly ask all classes in London to spend a few moments in considering the facts disclosed by these figures. While the people of London make use of the hospitals in ever-increasing numbers, the figures prove that they are not ready to make an adequate return for the benefits received, but are content to trust to the dead hand to make up any deficiency; and unless this lamentable lassitude in our charity gives place to more honest endeavour, the ultimate result is not far to seek. In London to-day there are some hospitals unable to take in the patients asking for admission, sometimes because the hospital is not large enough to cope with the district it serves; sometimes because beds are closed for want of funds. Look at this fact from whatever point we will, it is one to make us ashamed. We boast of being citizens of this rich and powerful centre of the greatest Empire the world has known. Yet we allow ourselves to remain in imminent danger of the reproach that in spite of our riches, in spite of our power, we grudge the help our suffering and less prosperous brethren need. After all, it is only a very small sacrifice on the part of each individual that is required, and to plead inability is in ninety-nine cases out of a hundred absolute dishonesty. You cannot afford to give a shilling or two to the hospitals, but you never think twice about giving half-a-crown, probably many times a year, for a place in the pit of a theatre. We can all do something. It is our duty; it should be our pleasure. Whether we like it or not, the care of the sick is entrusted to us, and who can be callous enough to reject this trust?

### WHAT EACH INSTITUTION SPECIALLY NEEDS.

**Royal London Ophthalmic Hospital.**—Only 70 beds out of 138 available (see p. 25).

**Royal Orthopædic Hospital.**—To be rebuilt on another site (see p. 7).

**Royal Sea Bathing Hospital, Margate.**—Deficit for 1901, £1,427. Reliable income less than expenditure by £5,768 (see p. 8).

**Royal Westminster Ophthalmic Hospital.**—Debt of £10,000 for reconstruction to be paid.

**St. George's Hospital.**—Expenditure exceeds income by £13,000 (see p. 27).

**St. Mark's Hospital for Fistula.**—£1,000 annual subscription required (see p. 7).

**St. Mary's Hospital.**—Wants £20,000 to complete and furnish new wing (see p. 4).

**St. Saviour's Hospital.**—Assist ladies of limited means (see p. 28).

**Samaritan Free Hospital.**—Deficit 1901, £400. Additional £4,300 required to meet expenditure (see p. 6).

**University College Hospital.** — Rebuilding (see p. 8).

**West End Hospital for Paralysis.**—More accommodation needed (see p. 24).

**Westminster Hospital.**—About £13,000 required from voluntary sources (see p. 5).

### Coronation Hospital Sunday.

### OUR GUESTS AND OUR HOSPITALS.

Hospital Sunday in Coronation Year will be memorable from the fact that many of the metropolitan congregations assembled in places of worship on that day will include residents in many parts of the Empire. They have come to do honour to the King and to take part in the rejoicings of the British nation and people. They will represent the intelligence and also the prosperity of the populations of every part of the British Empire. Amongst them will be some, at any rate, who are familiar with the Hospital Sunday movement which has spread to a few cities in our colonies and is likely one day to be universal. Naturally our brothers and sisters from beyond the seas may desire to mark the occasion by a special gift, and the King wishes it to be known that he desires each of his subjects to express their loyalty by combining with him to secure the necessary financial strength for our hospitals.

The true secret of England's strength is the English home. All that is best in our common humanity is blended into that glow of feeling which finds its centre and hope and joy in home. The war, now happily concluded, has proved once again that England is regarded as Home by every member of the race, no matter in what part of the British Empire his lot may be cast. For this reason we hope that the preachers will enforce this fact, for then we have little doubt that the yield on Coronation Hospital Sunday will be a record one. In the ordinary way Londoners do support their hospitals, but this year, led by the King in person, the representatives of every part of the Empire and nation who may be present in the metropolis will wish to prove their loyalty and to express thankfulness on so joyful an occasion as Coronation Hospital Sunday by giving in the churches or sending something to the Lord Mayor at the Mansion House, as treasurer of the Hospital Sunday Fund.

It may be said that London hospitals should be supported by Londoners alone, and that for this reason preachers in metropolitan churches ought not to appeal to the people who reside in the provinces or in the colonies. This objection will not stand the test of examination. London is the centre and metropolis of the Empire. It so becomes the trysting-place of the best brains and enterprise and the centre of the wealth of the nation. In London the majority of the medical men and nurses who minister to the sick and suffering throughout the country receive their training, and become fitted for their work. But London is more than this, for it has become to the Empire the Mecca of the sick. Whenever an obscure case of disease occurs, when the local medical attendants are in doubt or difficulty, when the friends of the patient become anxious, their first thought and wish is that the sufferer shall be sent to London, because there, if anywhere, can be brought to bear upon his malady the latest scientific developments and the greatest technical skill which the medical profession can afford. It follows that everybody, directly or indirectly, owes a duty to the London hospitals; and, as a matter of mere self-defence, it is of the first importance to everybody, apart altogether from their place of residence, to see that the London hospitals and everything connected with them should be maintained in the highest state of efficiency. This, the Coronation Year, is a very special year in the history of our nation; let us make Hospital Sunday the most memorable day in it by our free-will offerings to the sick.

### Where Figures Fail.

There are things which cannot be expressed in figures. What seems a little skirmish, so far as the telegraphic total of the killed and wounded is concerned, may be the turning-point of a campaign, and in the same way when we attempt, as we naturally do attempt, to tot up the work of the hospitals, and to express in the form of a balance-sheet the great work they have done, we are met with a great sense of failure. The figures do not express what we want to say.

If it could be done one would be glad to strike a balance between suffering and sorrow on the one side and charity and good works on the other; and, when the year was over, to close one's books with some sort of knowledge of how we stood—as to how far we had failed in meeting the evil with the good, and how much more we had to do before we could feel satisfied that we had performed our duty to our fellow man. Unfortunately this cannot be. Misery and sickness refuse to be assessed in pounds and shillings, and even charity though given, maybe, in cheques and sovereigns, cannot, so far as results are concerned, be estimated by such gross measures. Not only does the widow's mite stand for much, as a good deed well done, but it sometimes, if given just at the nick of time, may stand for more in the way of actual good service to the recipient than a far larger sum offered in a more formal manner. Hence the whole problem stands outside arithmetic. Still, one must have some sort of a basis on which to go, and it is satisfactory to find on referring to other columns of this our Coronation Hospital Sunday Supplement that so many thousands of in-patients have been received, that so many tens of thousands of out-patients have been treated, and that so many hundreds of thousands of golden sovereigns have been collected in the name of charity and spent on the support of hospitals. And to some this will be the measure of the good work. Let us, however, be a little more imaginative; let us think what one cure means; let us turn back to our own personal experiences, and ask what one life saved means to one family, and then try to multiply by the necessary thousands the grief and misery which existed in that one family while recovery was a matter of doubt, and the joy which was experienced when at last it was assured. Let us endeavour thus to form some conception of the sum total of such joys and sorrows, all of which hinge upon the successful conduct of our hospitals, and when we fail, as we must do, let us remember that our very failure shows how inconceivably great is the direct and personal beneficence of the hospitals of the metropolis whose claims upon the charitable we now urge in connection with the Hospital Sunday Fund.

### NEARLY TWO MILLION SUFFERERS HELPED BY THE HOSPITALS.

### A SINGLE YEAR'S ROLL-CALL OF THE SICK.

How many patients are annually treated in the London Hospitals ? The cases which we have sorted out under the various headings comprise those treated at the voluntary hospitals and dispensaries of London—together with the endowed hospitals of St. Bartholomew's, Guy's and St. Thomas's—and the hospitals of the Metropolitan Asylums Board. Altogether they reach the immense total of *one million nine hundred and sixteen thousand seven hundred and sixty-nine patients*; and dividing these into men, women and children, we arrive at the following result:—That of the 1,916,769 patients, 740,783 were men, 644,033 were women, and 531,953 were children.

884,702.
Surgical Patients.

**Patients Suffering from Surgical Diseases.**—Of the whole number of patients received by the hospitals, *eight hundred and eighty-four thousand seven hundred and two* required surgical treatment. Let us clearly understand what is meant by "surgical diseases." They include not only all accidents such as broken bones, fractured skulls, mangled limbs and all manner of displacements and crushings of sensitive parts and organs, but also abscesses, ulcerations, cancers, and tumours of all kinds ; in short all those injuries which may be produced by accident or pathological process, and which may be dealt with either by hand or instrument. That such a vast number of persons in London suffer from one or more of the injuries thus briefly summarised is not easy to realise. Perhaps it may be brought home to us in a measure if we remember the great army we have sent to fight our battles in South Africa, and compare its numerical strength with this immense army of 884,702 sufferers.

654,262.
Medical Patients.

**Patients Suffering from Medical Diseases.**—*Six hundred and fifty-four thousand two hundred and sixty-two* persons received medical treatment. By medical diseases is meant those diseases which are situated either as to their source and origin or in their entirety in one or the other of the three great cavities of the body. They include rheumatic fever, pneumonia, pleurisy, bronchitis, diseases of the stomach, bowels, liver, kidney, bladder, and pancreas, every kind of heart disease, many forms of brain injury, dyspepsia, constipation, most nervous diseases, and other ailments, many of them serious and many of them dangerous to life, or at least to the useful existence of the individual. Remember that most of these diseases are out of sight, that the diagnosis of their nature and extent, and the successful treatment of them, is dependent on the doctor's scientific knowledge, and then try and realise that in the hospitals of London over 654,000 persons received treatment at the hands of the foremost physicians of the day, free of cost to the patients themselves. Surely when the hospitals plead for help to us who are in health our answer should be a generous one. Good health is a free gift, and since we have received so lavishly we ought certainly, as a thankoffering, to give liberally.

**Patients Suffering from Eye Affections.**—*One hundred and twenty thousand seven hundred and seventy-five* persons were treated in the special departments of the general hospitals or by the ophthalmic hospitals of London. The statement that, apart from the blind, nearly an eighth of a million persons have in one year been treated for diseases of the eye in various forms should come home with peculiar force to those who are blessed with sight, and who, too seldom we fear, give one thought to what the loss of it would mean to them. It is certain that very many of these cases must have entailed terrible suffering, and many doubtless would have terminated in total loss of sight but for the skilful treatment they have received at the hospitals. Who can say how many have been saved from becoming practically helpless in the world ?

120,775.
Eye.

### THE ROLL-CALL OF THE SICK.—*Continued.*

**Patients Treated at Special Hospitals for Children.**—*One hundred and eighteen thousand two hundred and fifty-two* children were sent from homes where they could not be properly attended to for treatment in the hospitals. To see the small face pinched and pain-marked, instead of sunny and dimpled with smiles, the tiny limbs inert and still which should be full of joyous movement and new life, and to hear a low wail of agony from lips which were formed for childish prattle, must pierce the heart of every man and woman worthy the name. Parents who are happy in the knowledge of the noisy nursery at home, whose very lives echo with the pleasant pattering of little feet, cannot withhold their hands. When the children's hospitals appeal for funds to carry on the work of restoring to health and strength the little ones of this great city, the children who will one day take our place as the workers in this land of ours, let the children who are strong and well give to those who are ill and weak.

118,252.
Children.

**Diseases of Women and Motherhood.**—*Eighty thousand five hundred and sixty-four women* were treated for the special diseases of women at the metropolitan voluntary hospitals, the greater portion attending the hospitals for women and the lying-in institutions. Apart from the diseases to which all are liable, women have to face others peculiar to their sex entailing great suffering, resulting often in permanent or partial disablement. Here it is not only our sympathy which is appealed to, but our patriotism as well. Here there is an actual demand for the payment of a debt we most justly owe. The very heart and strength of the nation lies in the home life, and the soul of the home life is the woman—the mother. The majority of us are to-day what we are because of the influences brought to bear upon us in the home.

80,564.
Women.

**Patients Suffering from Diseases of the Ear and Throat.**—At the special hospitals or special departments devoted to these diseases *forty-five thousand six hundred and thirteen persons* were treated. It is startling to find that so many persons required treatment in the special hospitals for ear, nose, and throat. The affections and diseases of these organs, which are intimately connected, involve temporary and often permanent impairment of hearing, swallowing, and breathing. These functions are performed with so little effort on our part that, unless experience has taught us, it is difficult to understand what it would mean to us if we suddenly had to suffer from one or other of these affections.

45,613.
Ear and Throat.

**Patients Suffering from Consumption.**—*Forty-three thousand four hundred and sixty-seven* patients suffering from phthisis or consumption were treated at the consumption hospitals of London during the year. The very word consumption makes us afraid. There are few of us who have not seen something of its ravages, of its cruelty. Truly may consumption be called the curse of our climate. It respects neither persons nor estate, neither rich nor poor, the old or the young.

43,467.
Consumption.

**Patients Suffering from Diseases of the Skin.**—During the year *thirty-five thousand and seventy-two* persons were treated for skin diseases in London. It is, perhaps, more difficult to bring home to people the claim which sufferers from skin diseases have upon their sympathy than it is in any of the other classes of disease which we are considering. There is not, as a rule, the pain, nor the danger to life, nor even such risk of permanent disablement as is the case with many of the others; but let us remember what the result would be were there no hospitals for the sufferers to go to.

35,072.
Skin.

**Patients Suffering from Paralysis and Diseases of the Nerves.**—*Fifteen thousand six hundred and eighteen persons* stricken with paralysis and diseases of the brain received treatment at the hospitals devoted to these maladies. To workers busy with hand and brain these sufferers must particularly appeal. It is impossible to disassociate nervous breakdown from the toil and hurry of existence, especially in a vast centre like London. It is appalling to think that at any moment any one of us may be struck down suddenly, perhaps without the slightest warning. No disease is more sudden than paralysis, surely none more pitiful.

15,618.
Paralysis.

**Patients Suffering from Fever.**—The number of persons who suffered from various forms of fever during the year was 13,653. This figure is however a misleading one because the term fever includes much besides the class of fevers which are usually removed to these hospitals. Measles, for instance, prevails in London to such an extent that more deaths occur from it than from scarlet fever. The excellent service rendered by the London Fever Hospital entitles it to the gratitude of all householders.

13,653.
Fever.

So this great army of sufferers, numbering close upon two millions, claim our sympathy and our help year by year. To the strong, to those in health who are able to provide for those dependent upon them, to those who know what ill-health means, who have suffered from disease of one kind or another, and who, either in the hospital or under the skill and care of the doctors and nurses trained in the hospital, have been restored to health and usefulness, we confidently appeal on behalf of the London hospitals.

### WORKERS ON CORONATION HOSPITAL SUNDAY.

### THE PREACHERS.

IT would be difficult to exaggerate the recognition which the hospitals owe to the pulpit. Medicine and the Church have ever been in close alliance, and whereas those preachers who make a point of doing their utmost for the hospitals on Hospital Sunday are entitled to gratitude, all the clergy and ministers of religion must have opportunities during each year of receiving direct benefits from the hospitals through the Hospital Sunday Fund for one or more of their flock. To say this is but to balance the account, but to-day we do not wish to dwell upon the business aspect of the question. It may be difficult enough for preachers sometimes to find subjects, so that Hospital Sunday does certainly quicken the speech by firing the imagination of the majority, at any rate, of the men who sit down and try to realise what even one hour's work in any single one of our great hospitals in London to-day means to the population as a whole and to individual sufferers especially. If there should be any who desire a text which will fix attention and drive home palpable truths, we would suggest volcanoes and hospitals. The Martinique disaster, which has appalled the whole world by its suddenness and horror, is but typical of a state of affairs which exists in every community everywhere. The people of Martinique resident in the town of St. Pierre on either side the mountain, assured themselves no doubt when the mountain became disturbed, noisy, and threatening, that it would pass away, and in any case it would not hurt them. Is not that the position of the overwhelming majority of people everywhere when they are well in regard to sickness and disease? Many of the hale say and hold: "It is not our affair that our neighbour is sick. We come of a healthy and long-lived family. We have never known what a day's illness is since we can remember anything." And it is sad but true that the better the health of the individual the more difficult is it to make that individual realise what sickness means to his less fortunate neighbour, or even to a member of his own household, or of one of his own children. What difference is there between the hale who takes up the attitude—and it is the general attitude—which we have here stated, and the residents at St. Pierre on the eve of the eruption? Yet who of all the hale can tell how many hours will pass before illness and disease may effect for themselves individually everything that the volcano did for the people who perished in Martinique?

### THE PRESS.

It is, of course, a fact that the position of the editor of a Metropolitan paper in these days is one of intense difficulty, owing to the circumstance that the amount of copy which comes to him by telegraph alone may on some days be sufficient to more than fill the whole newspaper. Yet he has to find room for all kinds of topics—imperial, national, metropolitan, social, scientific, and general. No wonder if some interests are seldom, or never, noticed. It is but just, however, to recognise the generosity with which the editors of the London papers co-operate whenever possible to help the London hospitals. We have long felt that if the editors could see their way to devote a certain amount of space each day during the week immediately preceding Hospital Sunday in each year to hospital subjects, so as to quicken public attention and rouse public interest, they would be able to claim this service as a valid excuse for not ordinarily giving space to hospital appeals, and at the same time render a greater service to the cause of the hospitals than probably in any other way. To-day in the metropolis of the Empire, before any single topic can be impressed upon its inhabitants, it is essential that it should be noticed not once or twice but three or four times continuously, or nearly continuously, within a very limited number of days. We therefore venture to hope that our friends and *confrères* in the Metropolitan daily press will kindly consider this suggestion of ours as to a hospital week for press purposes on the lines we have indicated. If it can be adopted the results may surprise the most optimistic of journalists.

### THE LAITY.

In connection with Hospital Sunday the laity can and ought to welcome the opportunity of rendering two services to the hospitals. First of all they can co-operate with the clergy and ministers of religion by offering to do a certain proportion of the circular work by sending out letters to absent members of the congregation asking them to send their contributions to be placed in the plates on Hospital Sunday. Secondly, every well-organised and intelligent congregation nowadays should have a Hospital Society. That society should consist of a chairman and honorary secretary who should make it their business to interest members of the congregation in hospitals and their work. We have set forth the objects of such a society already on another place (page 11), and need not repeat them here. Our point, however, is that the laity, and especially the younger laity, with the Church officers, would find it of material service to the poorer members of their congregation if they would take this suggestion to heart. No congregation can be said, so far as its members are concerned, to show that they are sincere and convinced members of a church when they fail to ascertain and satisfy themselves that adequate means exist whereby every poor member and neighbour can and does receive the medical aid and nursing care which he requires when ill. To succeed, nay, to justify the right of the individual to live, in a highly civilised community, it is essential that he or she shall have sympathy. Without sympathy life is mostly selfishness and obesity, a distorted and repellant thing which produces animals rather than intelligent men and women. We therefore hope that Hospital Sunday in the year of the Coronation may at any rate promote an alteration in regard to the attitude of the majority of the laity towards the Hospital Sunday Fund, towards the hospitals themselves and the sick who use them, resulting in the institution this year of a large number of Hospital Societies in connection with the individual congregations which make collections on Hospital Sunday. The humanising influence of such a new departure upon the whole population of London must work for good, and may ultimately lead to life in cities being better for all classes by making it possible that we may all be less selfish and more neighbourly.

### A YEAR'S WORK IN THE HOSPITALS OF LONDON, 1902.

### NEWINGTON AND SOUTH DISTRICT.

Comprising Battersea, Wandsworth, Tooting, Balham, Streatham, Brixton, Lambeth, Newington, Southwark, Bermondsey, Camberwell, Greenwich, Deptford, Lewisham, Blackheath, Woolwich, &c.

| No. of Beds. | No. of Beds Daily Occupied. | HOSPITALS. | In-patients. | Out-patients. | Total Expenditure. | Income. | | | Total Income. | Legacies not included in previous column. |
|---|---|---|---|---|---|---|---|---|---|---|
| | | | | | | Charitable. | Proprietary. | Patients' Payments. | | |
| | | | | | £ | £ | £ | £ | £ | £ |
| 652 | 470 | Guy's ... ... ... ... | 7,326 | 104,316 | 89,130 | 15,127 | 56,756 | 2,796 | 74,679 | 10,927 |
| 11 | 8 | Phillips' Memorial Homœopathic | 128 | 961 | 915 | 451 | 180 | 299 | 930 | ... |
| 25 | 20 | Miller ... ... ... ... | 352 | 17,639 | 3,723 | 3,294 | 395 | ... | 3,689 | 180 |
| 586 | 474 | St. Thomas's ... ... ... | 6,605 | 62,861 | 75,440 | 7,180 | 47,902 | 234 | 55,316 | 9,773 |
| 269 | 236 | Seamen's ... ... ... ... | 2,634 | 22,542 | 21,909 | 9,021 | 4,205 | 896 | 14,122 | 771 |
| 66 | 61 | Evelina, for Children ... | 981 | 6,677 | 7,656 | 1,687 | 3,438 | 92 | 5,217 | 60,384 |
| 57 | 46 | Home for Sick Children... ... | 217 | 1,560 | 1,802 | 1,178 | 243 | 284 | 1,705 | 520 |
| 24 | 20 | General Lying-in... ... ... | 497 | 1,887 | 3,575 | 703 | 2,622 | ... | 3,325 | ... |
| 36 | 15 | Clapham Maternity & Dispensary | 350 | 5,536 | 3,426 | 459 | 960 | 946 | 2,365 | ... |
| 54 | 50 | Royal, for Children and Women . | 488 | 18,044 | 4,705 | 2,634 | 1,097 | 250 | 3,981 | 340 |
| 42 | 33 | Royal Eye ... ... ... ... | 605 | 17,840 | 3,426 | 2,491 | 182 | 578 | 3,251 | 203 |
| 11 | 9 | Eltham Cottage ... ... ... | 145 | ... | 716 | 549 | 29 | 195 | 773 | ... |
| 30 | 11 | Beckenham Cottage ... ... | 191 | ... | 891 | 547 | 6 | 125 | 678 | 25 |
| 18 | 11 | Blackheath Cottage ... ... | 167 | 1,066 | 911 | 767 | 33 | 108 | 908 | 100 |
| 32 | 21 | Bromley Cottage ... ... ... | 351 | ... | 1,465 | 1,029 | 248 | 169 | 1,446 | 500 |
| 14 | 9 | Chislehurst, &c., Cottage ... | 119 | ... | 658 | 485 | 57 | 143 | 685 | ... |
| 14 | 7 | Sidcup Cottage ... ... ... | 148 | 70 | 611 | 457 | 13 | 142 | 612 | ... |
| 7 | 5 | Shortlands... ... ... ... | 83 | ... | 176 | 190 | 1 | 2 | 193 | ... |
| 17 | 13 | Livingstone Cottage ... ... | 157 | ... | 1,001 | 984 | 24 | 76 | 1,084 | ... |
| 10 | 7 | Woolwich and Plumstead Cottage | 73 | 19 | 501 | 426 | 11 | 96 | 533 | ... |
| 9 | 7 | St. Francis ... ... ... | 158 | 10,009 | 1,433 | 1,005 | 60 | 366 | 1,431 | ... |
| 41 | 23 | St. John's, Lewisham ... ... | 322 | ... | 2,334 | 747 | 8 | 282 | 1,037 | ... |
| 2,025 | 1,556 | | 22,097 | 271,027 | 225,252 | 51,411 | 118,470 | 8,079 | 177,960 | 83,723 |
| | | DISPENSARIES. | | | | | | | | |
| ... | ... | Battersea Provident ... ... | ... | 19,282 | 3,370 | 90 | 69 | 3,262 | 3,421 | ... |
| ... | ... | Blackfriars, Provident ... ... | ... | 600 | 280 | 87 | ... | 157 | 244 | ... |
| ... | ... | Brixton, &c. ... ... ... | ... | 3,963 | 640 | 482 | 36 | 64 | 582 | ... |
| ... | ... | Camberwell Provident ... ... | ... | 6,965 | 1,846 | 342 | 189 | 1,403 | 1,934 | 10 |
| ... | ... | Clapham ... ... ... ... | ... | 1,363 | 337 | 240 | 29 | 105 | 374 | ... |
| ... | ... | Deptford Medical Mission ... | ... | 2,917 | 339 | 246 | 31 | 69 | 346 | ... |
| ... | ... | East Dulwich Provident ... | ... | 4,953 | 973 | 122 | 2 | 834 | 958 | ... |
| ... | ... | Forest Hill ... ... ... | ... | 2,121 | 677 | 224 | 13 | 453 | 690 | 45 |
| ... | ... | Greenwich Provident ... ... | ... | 2,933 | 642 | 57 | 16 | 595 | 668 | ... |
| ... | ... | Royal South London ... ... | ... | 4,142 | 648 | 498 | 112 | ... | 610 | ... |
| ... | ... | South Lambeth, &c. ... ... | ... | 2,109 | 658 | 345 | 14 | 160 | 519 | ... |
| ... | ... | Walworth Provident ... ... | ... | 827 | 284 | 46 | 30 | 164 | 240 | ... |
| ... | ... | Wandsworth Common ... ... | ... | 951 | 221 | 49 | ... | 172 | 221 | ... |
| ... | ... | Woolwich, &c., Provident ... | ... | 2,005 | 407 | 28 | ... | 300 | 328 | ... |
| 2,025 | 1,556 | | 22,097 | 326,158 | 236,574 | 54,267 | 119,011 | 15,817 | 189,095 | 83,778 |

### WESTMINSTER DISTRICT.—Comprising Westminster City and Liberties.

| No. of Beds. | No. of Beds Daily Occupied. | HOSPITALS. | In-patients. | Out-patients. | Total Expenditure. | Charitable. | Proprietary. | Patients' Payments. | Total Income. | Legacies not included in previous column. |
|---|---|---|---|---|---|---|---|---|---|---|
| | | | | | £ | £ | £ | £ | £ | £ |
| 150 | 100 | Charing Cross ... ... ... | 1,584 | 13,820 | 24,394 | 10,726 | 2,689 | 107 | 13,522 | 8,332 |
| 223 | 184 | King's College ... ... ... | 2,582 | 20,471 | 20,372 | 9,143 | 4,255 | 146 | 13,544 | 9,526 |
| 213 | 179 | Westminster ... ... ... | 2,743 | 22,290 | 20,215 | 16,425 | 2,867 | 63 | 19,355 | 14,361 |
| 152 | 144 | Ventnor, for Consumption ... | 776 | ... | 14,360 | 7,179 | 2,248 | 4,049 | 13,476 | 6,712 |
| 23 | 18 | Grosvenor, for Women & Children | 210 | 5,909 | 2,508 | 2,361 | 46 | 533 | 2,940 | 2,403 |
| 64 | 38 | Hospital for Women ... ... | 593 | 4,327 | 5,385 | 3,630 | 253 | 643 | 4,526 | 405 |
| 25 | 13 | National, for Diseases of Heart... | 103 | 2,246 | 2,046 | 1,442 | 93 | 497 | 2,032 | ... |
| 36 | 26 | Royal Westminster Ophthalmic... | 436 | 6,640 | 2,165 | 2,322 | 708 | 45 | 3,075 | 63 |
| 50 | 39 | Royal Orthopædic ... ... | 255 | 1,060 | 2,849 | 1,254 | 384 | 242 | 1,880 | 115 |
| 9 | 7 | Royal Ear ... ... ... ... | 248 | 2,011 | 848 | 344 | ... | 552 | 896 | ... |
| ... | ... | Dental ... ... ... ... | ... | 39,737 | 2,794 | 2,574 | 239 | 257 | 3,070 | 1,742 |
| 27 | 19 | Gordon, for Fistula ... ... | 295 | 916 | 1,695 | 329 | ... | 1,324 | 1,653 | 90 |
| 27 | 20 | St. Peter's, for Stone ... ... | 490 | 4,402 | 4,252 | 1,164 | 329 | 2,184 | 3,677 | ... |
| 50 | 33 | St. John's, for Skin ... ... | 239 | 7,403 | 4,285 | 1,533 | 88 | 2,348 | 3,969 | ... |
| 36 | 31 | Hospital for Diseases of Throat... | 702 | 10,895 | 5,686 | 979 | 34 | 3,651 | 4,664 | 1,034 |
| 1,075 | 844 | | 11,256 | 142,127 | 113,854 | 61,405 | 14,233 | 16,641 | 92,279 | 44,783 |
| | | DISPENSARIES. | | | | | | | | |
| ... | ... | Public ... ... ... ... | ... | 2,170 | 652 | 434 | 220 | ... | 654 | ... |
| ... | ... | St. George and St. James ... | ... | 3,448 | 546 | 392 | 8 | 33 | 433 | ... |
| ... | ... | St. George's, Hanover Square ... | ... | 2,129 | 528 | 403 | ... | 153 | 556 | ... |
| ... | ... | Western ... ... ... ... | ... | 9,852 | 1,499 | 321 | 419 | 690 | 1,430 | ... |
| ... | ... | Westminster General ... ... | ... | 6,137 | 696 | 372 | 195 | 117 | 684 | ... |
| 1,075 | 844 | | 11,256 | 165,861 | 117,775 | 63,327 | 15,075 | 17,634 | 96,036 | 44,783 |

### ST. MARYLEBONE AND WEST CENTRAL DISTRICT.

Comprising St. Marylebone, St. John's Wood, Bloomsbury, Holborn, &c.

| No. of Beds. | No. of Beds Daily Occupied. | HOSPITALS. | In-patients. | Out-patients. | Total Expenditure. | Income. | | | Total Income. | Legacies not included in previous column |
|---|---|---|---|---|---|---|---|---|---|---|
| | | | | | | Charitable. | Proprietary. | Patients' Payments. | | |
| | | | | | £ | £ | £ | £ | £ | £ |
| 70 | 52 | French ... ... ... ... | 820 | 4,667 | 4,065 | 4,026 | 20 | ... | 4,046 | 1,590 |
| 50 | 28 | Italian ... ... ... ... | 520 | 8,103 | 2,258 | 2,133 | 266 | ... | 2,399 | ... |
| 103 | 79 | London Homœopathic ... ... | 1,092 | 21,822 | 11,090 | 3,138 | 3,212 | 918 | 7,268 | 684 |
| 59 | 51 | SS. John and Elizabeth ... ... | 95 | ... | 3,579 | 3,185 | 512 | 788 | 4,485 | 121 |
| 381 | 297 | The Middlesex ... ... ... | 3,777 | 45,084 | 43,768 | 10,112 | 10,941 | ... | 21,053 | 19,596 |
| 70 | 56 | Alexandra for Children ... ... | 112 | 360 | 4,714 | 4,093 | 265 | 408 | 4,766 | ... |
| 30 | 29 | Hospital for Incurable Children | 31 | ... | 1,331 | 1,001 | 104 | 445 | 1,550 | 511 |
| 252 | 172 | Hospital for Sick Children ... | 2,314 | 24,670 | 20,509 | 8,224 | 4,132 | ... | 12,356 | 3,697 |
| 20 | 16 | British Lying-in ... ... ... | 467 | 616 | 2,585 | 652 | 1,885 | 89 | 2,626 | ... |
| 74 | 56 | Queen Charlotte's Lying-In ... | 1,407 | 1,026 | 5,520 | 4,034 | 393 | 118 | 4,545 | 1,575 |
| 50 | 44 | New Hospital for Women ... | 573 | 14,936 | 5,372 | 3,054 | 1,010 | 1,544 | 5,608 | 45 |
| 47 | 45 | Samaritan Free ... ... ... | 486 | 7,227 | 5,882 | 4,548 | 286 | ... | 4,834 | 545 |
| 200 | 177 | National for the Paralysed, &c.... | 952 | 6,150 | 16,725 | 6,377 | 1,660 | 2,802 | 10,839 | 2,231 |
| Clo | sed | Hospital for Epilepsy, &c. ... | ... | 595 | 854 | 659 | 10 | 172 | 841 | 350 |
| 50 | 25 | West End, for Epilepsy, &c. ... | 261 | 3,481 | 4,237 | 2,898 | 102 | 520 | 3,520 | 200 |
| 28 | 17 | Central London Ophthalmic ... | 332 | 12,069 | 1,832 | 1,840 | 51 | ... | 1,891 | ... |
| 13 | 6 | Western Ophthalmic ... ... | 173 | 10,052 | 1,080 | 512 | 168 | 4 | 684 | 235 |
| 60 | 57 | National Orthopædic ... ... | 203 | 833 | 2,543 | 1,717 | 101 | 865 | 2,683 | 25 |
| 20 | 9 | Establishment for Gentlewomen. | 144 | ... | 2,576 | 827 | 72 | 886 | 1,785 | 500 |
| ... | ... | National Dental ... ... ... | ... | 18,485 | 1,820 | 426 | ... | 960 | 1,386 | ... |
| 16 | 9 | London Throat ... ... ... | 609 | 5,407 | 1,430 | 494 | ... | 1,023 | 1,517 | ... |
| 17 | 5 | Metropolitan Ear, Nose & Throat | 232 | 2,569 | 687 | 350 | ... | 312 | 662 | ... |
| 32 | 19 | Oxygen Hospital ... ... ... | 74 | ... | 969 | 382 | 19 | 398 | 799 | ... |
| 1,642 | 1,249 | | 14,674 | 188,152 | 145,426 | 64,682 | 25,209 | 12,252 | 102,143 | 31,405 |
| | | DISPENSARIES. | | | | | | | | |
| ... | ... | Bloomsbury Provident ... ... | ... | 789 | 282 | 30 | ... | 211 | 241 | ... |
| ... | ... | London Medical Mission ... | ... | 6,856 | 1,375 | 795 | 61 | 253 | 1,109 | 150 |
| ... | ... | Portland Town ... ... ... | ... | 1,334 | 165 | 123 | 14 | 10 | 147 | ... |
| ... | ... | St. John's Wood Provident ... | ... | 5,639 | 697 | 275 | 1 | 395 | 671 | 100 |
| ... | ... | St. Marylebone General ... ... | ... | 3,973 | 842 | 433 | 163 | 213 | 809 | 100 |
| ... | ... | Western General ... ... ... | ... | 15,894 | 1,357 | 1,573 | 26 | 59 | 1,658 | 625 |
| 1,642 | 1,249 | | 14,674 | 222,637 | 150,144 | 67,911 | 25,474 | 13,393 | 106,778 | 32,380 |

### STRATFORD AND EAST-END DISTRICT.

Comprising Bethnal Green, Tower Hamlets, West Ham, Whitechapel, Hackney, Stepney, Limehouse, Poplar, and the East.

| No. of Beds. | No. of Beds Daily Occupied. | HOSPITALS. | In-patients. | Out-patients. | Total Expenditure. | Income. | | | Total Income. | Legacies not included in previous column. |
|---|---|---|---|---|---|---|---|---|---|---|
| | | | | | | Charitable. | Proprietary. | Patients' Payments. | | |
| | | | | | £ | £ | £ | £ | £ | £ |
| 130 | 115 | German ... ... ... ... | 1,688 | 24,203 | 10,665 | 5,041 | 2,421 | 444 | 7,906 | 568 |
| 790 | 678 | London ... ... ... ... | 12,723 | 169,020 | 146,412 | 41,933 | 54,902 | 1,567 | 98,402 | 11,442 |
| 50 | 37 | Mildmay Mission Hospital ... | 515 | 9,850 | 3,769 | 2,587 | 571 | 56 | 3,214 | ... |
| 87 | 59 | Poplar ... ... ... ... | 1,235 | 20,959 | 12,202 | 10,616 | 1,100 | 101 | 11,817 | 687 |
| 60 | 46 | West Ham, &c. ... ... ... | 933 | 20,890 | 6,040 | 5,037 | 235 | ... | 5,272 | 2,769 |
| 34 | 23 | Walthamstow, &c. ... ... | 397 | 1,207 | 1,463 | 1,136 | 247 | ... | 1,383 | 90 |
| 164 | 120 | City of London for Dis. of the Chest | 966 | 9,678 | 11,487 | 8,141 | 310 | ... | 8,451 | 328 |
| 135 | 118 | East London for Children ... | 1,992 | 36,475 | 9,861 | 8,000 | 1,129 | ... | 9,129 | 1,060 |
| 35 | 30 | St. Mary's, Plaistow ... | 742 | 18,217 | 3,444 | 3,045 | 214 | 161 | 3,420 | 263 |
| 18 | 8 | East End Mothers' Home ... | 198 | 329 | 1,662 | 1,109 | 533 | 45 | 1,687 | 50 |
| 16 | 5 | Passmore Edwards Cottage, T'lb'ry | 83 | 432 | 646 | 768 | 23 | ... | 791 | ... |
| 13 | 10 | Canning Town Cottage ... ... | 130 | 1,427 | 1,021 | 480 | 288 | 198 | 966 | ... |
| 1,532 | 1,249 | | 21,602 | 312,687 | 208,672 | 87,893 | 61,973 | 2,572 | 152,438 | 17,257 |
| | | DISPENSARIES. | | | | | | | | |
| ... | ... | Eastern ... ... ... ... | ... | 8,113 | 872 | 179 | 278 | 284 | 741 | 10 |
| ... | ... | Hackney Provident ... ... | ... | 907 | 268 | 64 | ... | 199 | 263 | ... |
| ... | ... | London ... ... ... ... | ... | 2,463 | 416 | 123 | 277 | ... | 400 | ... |
| ... | ... | Queen Adelaide's ... ... ... | ... | 5,909 | 529 | 393 | 220 | ... | 613 | 10 |
| ... | ... | Tower Hamlets ... ... ... | ... | 4,700 | 615 | 288 | 22 | 166 | 476 | 250 |
| ... | ... | Whitechapel Provident ... ... | ... | 4,245 | 855 | 81 | ... | 746 | 827 | ... |
| ... | ... | Leman Street, Provident ... | ... | 1,000 | 510 | 278 | ... | 291 | 569 | ... |
| 1,532 | 1,249 | | 21,602 | 340,024 | 212,737 | 89,299 | 62,770 | 4,258 | 156,327 | 17,527 |

### ISLINGTON AND NORTH-WEST DISTRICT.

Comprising Islington, Holloway, Highbury, Hampstead, Highgate, St. Pancras, Stoke Newington, Tottenham, &c.

| No. of Beds. | No. of Beds Daily Occupied. | HOSPITALS. | In-patients. | Out-patients. | Total Expenditure. | Income. Charitable. | Income. Proprietary. | Income. Patients' Payments. | Total Income. | Legacies not included in previous column. |
|---|---|---|---|---|---|---|---|---|---|---|
| | | | | | £ | £ | £ | £ | £ | £ |
| 159 | 134 | Great Northern Central ... ... | 1,811 | 26,632 | 13,502 | 6,745 | 1,161 | 517 | 8,423 | 3,223 |
| 32 | 24 | Hampstead Hospital ... ... | 379 | 3,965 | 2,721 | 1,730 | 49 | 362 | 2,141 | ... |
| 120 | 84 | London Temperance ... ... | 1,299 | 21,853 | 12,616 | 4,343 | 1,889 | 300 | 6,532 | 4,820 |
| 53 | 47 | North-West London ... ... | 639 | 19,556 | 4,852 | 2,676 | 226 | 39 | 2,941 | 226 |
| 73 | 45 | Tottenham Training ... ... | 713 | 14,789 | 6,120 | 4,396 | 1,248 | ... | 5,644 | ... |
| 185 | 164 | University College ... ... | 2,695 | 42,569 | 25,178 | 8,580 | 5,315 | 24 | 13,919 | 13,683 |
| 100 | 97 | Mount Vernon for Consumption | 578 | 4,499 | 9,730 | 7,757 | 134 | 4 | 7,895 | 2,835 |
| 200 | 83 | London Fever ... ... ... | 810 | ... | 12,768 | 7,824 | 1,709 | 2,007 | 11,540 | 1,033 |
| 28 | 15 | Invalid Asylum ... ... ... | 200 | ... | 803 | 384 | 177 | 159 | 720 | ... |
| 14 | 13 | Children's Home Hospital, Barnet | 52 | ... | 507 | 560 | ... | 83 | 643 | ... |
| 14 | 11 | Enfield Cottage ... ... ... | 156 | ... | 602 | 584 | 32 | 11 | 627 | ... |
| 30 | 20 | Memorial Cottage, Mildmay ... | 179 | ... | 1,267 | 455 | 968 | 163 | 1,586 | ... |
| 27 | 17 | St. Saviour's Home ... ... | 130 | ... | 2,127 | 1,243 | 11 | 824 | 2,078 | 25 |
| 42 | 31 | Friedenheim Hospital ... ... | 110 | ... | 3,841 | 4,982 | 130 | 153 | 5,265 | 10 |
| 28 | 19 | St. Monica's, Brondesbury ... | 46 | ... | 1,691 | 1,162 | 265 | 270 | 1,697 | 535 |
| 25 | 18 | Willesden Cottage ... ... | 229 | ... | 1,300 | 1,288 | 57 | 74 | 1,419 | 667 |
| 20 | 8 | Bushey Heath Cottage ... ... | 102 | 55 | 849 | 706 | 55 | 136 | 897 | ... |
| 20 | 18 | Santa Claus Home ... ... | 29 | ... | 704 | 575 | 13 | 73 | 661 | ... |
| 1,170 | 848 | | 10,157 | 134,118 | 101,178 | 55,990 | 13,439 | 5,199 | 74,628 | 27,057 |
| | | DISPENSARIES. | | | | | | | | |
| ... | ... | Camden Provident ... ... | ... | 1,085 | 289 | 20 | ... | 256 | 276 | ... |
| ... | ... | Hampstead Provident ... ... | ... | 11,529 | 959 | 189 | 23 | 756 | 968 | ... |
| ... | ... | Holloway and North Islington ... | ... | 3,496 | 805 | 250 | 42 | 295 | 587 | ... |
| ... | ... | Islington ... ... ... ... | ... | 11,626 | 877 | 296 | 17 | 563 | 876 | ... |
| ... | ... | St. Pancras and Northern ... | ... | 1,531 | 535 | 225 | 149 | 100 | 474 | 100 |
| ... | ... | Stamford Hill, &c. ... ... | ... | 7,641 | 805 | 530 | 162 | ... | 692 | 10 |
| ... | ... | Childs' Hill, Provident ... ... | ... | 482 | 326 | 30 | 13 | 285 | 328 | ... |
| 1,170 | 848 | | 10,157 | 171,508 | 105,774 | 57,530 | 13,845 | 7,454 | 78,829 | 27,167 |

### KENSINGTON AND WEST DISTRICT.

Comprising Kensington, Paddington, Bayswater, Kilburn, Chelsea, Brompton, Fulham, Hammersmith, Chiswick, Brentford, Acton, Ealing, &c.

| No. of Beds. | No. of Beds Daily Occupied. | HOSPITALS. | In-patients. | Out-patients. | Total Expenditure. | Income. Charitable. | Income. Proprietary. | Income. Patients' Payments. | Total Income. | Legacies not included in previous column. |
|---|---|---|---|---|---|---|---|---|---|---|
| | | | | | £ | £ | £ | £ | £ | £ |
| 9 | 8 | Queen's Jubilee ... ... ... | 135 | 6,626 | 1,152 | 1,035 | 20 | ... | 1,055 | 1,000 |
| 351 | 300 | St. George's ... ... ... | 4,448 | 31,843 | 44,934 | 13,095 | 15,528 | 63 | 28,686 | 4,740 |
| 281 | 250 | St. Mary's ... ... ... ... | 3,812 | 43,681 | 28,809 | 10,997 | 2,382 | ... | 13,379 | 9,777 |
| 133 | 123 | West London ... ... ... | 2,126 | 32,176 | 10,452 | 6,651 | 787 | ... | 7,438 | 4,812 |
| 321 | 248 | Hospital for Consumption ... | 1,241 | 7,124 | 31,852 | 11,986 | 6,847 | ... | 18,833 | 5,095 |
| 16 | 15 | Belgrave, for Children ... ... | 251 | 5,692 | 1,257 | 520 | 94 | ... | 614 | 188 |
| 50 | 50 | Cheyne, for Sick & Incurable Chldn | 24 | ... | 2,854 | 1,881 | 548 | 356 | 2,785 | 300 |
| 46 | 37 | Paddington Green, for Children | 592 | 16,203 | 4,312 | 2,982 | 192 | 325 | 3,499 | 125 |
| 92 | 74 | Victoria, for Children ... | 927 | 16,869 | 7,315 | 5,339 | 545 | 372 | 6,256 | 2,120 |
| 52 | 46 | Chelsea, for Women ... ... | 730 | 2,711 | 6,101 | 3,478 | 141 | 944 | 4,563 | 692 |
| 105 | 89 | Cancer ... ... ... ... | 704 | 1,604 | 12,732 | 7,213 | 4,697 | ... | 11,910 | 20,568 |
| 135 | 98 | Female Lock ... ... ... | 690 | ... | 5,010 | 3,940 | 30 | 2,637 | 6,607 | 1,948 |
| 16 | 8 | Epsom and Ewell Cottage ... | 117 | ... | 1,076 | 545 | 14 | 165 | 724 | ... |
| 30 | 18 | Reigate and Redhill Cottage ... | 266 | ... | 1,122 | 956 | ... | 192 | 1,148 | 100 |
| 16 | 8 | Wimbledon Cottage ... ... | 124 | ... | 644 | 503 | 12 | 96 | 611 | ... |
| 15 | 5 | Hounslow Cottage ... ... | 85 | 333 | 485 | 261 | 208 | 40 | 509 | ... |
| 1,668 | 1,377 | | 16,272 | 164,862 | 160,107 | 71,382 | 32,045 | 5,190 | 108,617 | 51,465 |
| | | DISPENSARIES. | | | | | | | | |
| ... | ... | Brompton Provident ... ... | ... | 869 | 359 | 88 | 93 | 180 | 361 | ... |
| ... | ... | Chelsea, &c. ... ... ... | ... | 3,770 | 648 | 309 | 171 | ... | 480 | ... |
| ... | ... | Chelsea Provident ... ... | ... | 531 | 254 | 30 | 17 | 162 | 209 | ... |
| ... | ... | Kensal Town Provident ... ... | ... | 678 | 295 | 29 | ... | 242 | 271 | ... |
| ... | ... | Kensington ... ... ... | ... | 3,189 | 951 | 840 | 59 | ... | 899 | 55 |
| ... | ... | Kilburn, Maida Vale ... ... | ... | 3,444 | 456 | 389 | 44 | ... | 433 | ... |
| ... | ... | Kilburn Provident ... ... | ... | 6,278 | 1,186 | 53 | 15 | 1,144 | 1,212 | ... |
| ... | ... | Notting Hill Provident ... ... | ... | 560 | 245 | 68 | ... | 132 | 200 | ... |
| ... | ... | Paddington Provident ... ... | ... | 2,913 | 534 | 138 | 25 | 342 | 505 | 10 |
| ... | ... | Royal Pimlico Provident... ... | ... | 1,163 | 884 | 340 | 15 | 538 | 893 | ... |
| ... | ... | Westbourne Provident ... ... | ... | 2,660 | 497 | 43 | 39 | 354 | 436 | ... |
| 1,668 | 1,377 | | 16,272 | 190,917 | 166,416 | 73,709 | 32,523 | 8,284 | 114,516 | 51,530 |

### CITY AND EAST CENTRAL DISTRICT.

Comprising the City, St. Luke's, Shoreditch, Finsbury, and Clerkenwell.

| No. of Beds. | No. of Beds Daily Occupied. | HOSPITALS. | In-patients. | Out-patients. | Total Expenditure. | Income. | | | Total Income. | Legacies not included in previous column. |
|---|---|---|---|---|---|---|---|---|---|---|
| | | | | | | Charitable. | Proprietary. | Patients' Payments. | | |
| | | | | | £ | £ | £ | £ | £ | £ |
| 111 | 86 | Metropolitan ... ... ... | 1,131 | 39,431 | 12,322 | 9,393 | 374 | 1,131 | 10,898 | 1,568 |
| 170 | 131 | Royal Free ... ... ... | 2,026 | 35,409 | 13,100 | 8,365 | 982 | ... | 9,347 | 4,890 |
| 80 | 57 | Royal, for Diseases of the Chest. | 709 | 6,875 | 10,486 | 8,964 | 171 | ... | 9,135 | 522 |
| 57 | 47 | North-Eastern, for Children ... | 722 | 19,116 | 6,484 | 4,153 | 290 | 681 | 5,124 | ... |
| 42 | 28 | City of London Lying-In ... | 650 | 2,051 | 3,358 | 652 | 4,215 | 1 | 4,868 | 25 |
| 45 | 33 | St. Mark's, for Fistula ... ... | 485 | 1,314 | 4,384 | 1,988 | 724 | ... | 2,712 | 544 |
| 138 | 64 | Royal London Ophthalmic ... | 1,866 | 31,258 | 11,396 | 7,371 | 545 | ... | 7,916 | 904 |
| 50 | 43 | City Orthopædic ... ... ... | 295 | 3,890 | 2,351 | 1,985 | 16 | ... | 2,001 | ... |
| 17 | 10 | Central London Throat and Ear. | 260 | 8,510 | 2,414 | 596 | 75 | 1,543 | 2,214 | ... |
| 710 | 499 | | 8,144 | 147,854 | 66,295 | 43,467 | 7,392 | 3,356 | 54,215 | 8,453 |
| | | DISPENSARIES. | | | | | | | | |
| ... | ... | City ... ... ... ... ... | ... | 6,813 | 1,086 | 1,198 | 97 | ... | 1,295 | 22 |
| ... | ... | City of London and East London | ... | 26,292 | 1,790 | 94 | 98 | 2,154 | 2,346 | ... |
| ... | ... | Farringdon General ... ... | ... | 2,160 | 561 | 315 | 2 | 193 | 510 | ... |
| ... | ... | Finsbury ... ... ... ... ... | ... | 12,894 | 958 | 357 | 175 | 274 | 806 | 138 |
| ... | ... | Metropolitan ... ... ... | ... | 5,388 | 824 | 406 | 123 | 257 | 786 | ... |
| ... | ... | Royal General ... ... ... | ... | 2,933 | 728 | 314 | 356 | 83 | 753 | ... |
| 710 | 499 | | 8,144 | 204,344 | 72,242 | 46,151 | 8,243 | 6,317 | 60,711 | 8,613 |

### THE METROPOLITAN HOSPITALS.—A SUMMARY OF THE WORK DONE IN 1901.

It will be seen from the following summary that the Voluntary Hospitals and medical Charities of London, during the twelve months ending December 31st, 1901, relieved over One million seven hundred and twenty-five thousand patients at a cost of £1,061,662. The Ordinary Income only amounted to £802,292, leaving a deficiency of £259,370 on the year's work. The legacies received in 1901 amounted to £265,778, being £56,552 more than the amount received in 1900.

| No. of Beds. | No. of Beds Daily Occupied. | HOSPITALS AND DISPENSARIES. | In-patients. | Out-patients. | Total Expenditure. | Income. | | | Total Income. | Legacies not included in previous column. |
|---|---|---|---|---|---|---|---|---|---|---|
| | | | | | | Charitable. | Proprietary. | Patients' Payments. | | |
| | | | | | £ | £ | £ | £ | £ | £ |
| 2,025 | 1,556 | Newington and South District... | 22,097 | 326,158 | 236,574 | 54,267 | 119,011 | 15,817 | 189,095 | 83,778 |
| 710 | 499 | City and East Central District... | 8,144 | 204,344 | 72,242 | 46,151 | 8,243 | 6,317 | 60,711 | 8,613 |
| 1,075 | 844 | Westminster District ... ... | 11,256 | 165,861 | 117,775 | 63,327 | 15,075 | 17,634 | 96,036 | 44,783 |
| 1,642 | 1,249 | St. Marylebone and West Central District ... ... | 14,674 | 222,637 | 150,144 | 67,911 | 25,474 | 13,393 | 106,778 | 32,380 |
| 1,668 | 1,377 | Kensington and West District ... | 16,272 | 190,917 | 166,416 | 73,709 | 32,523 | 8,284 | 114,516 | 51,530 |
| 1,170 | 848 | Islington & North-West District | 10,157 | 171,508 | 105,774 | 57,530 | 13,845 | 7,454 | 78,829 | 27,167 |
| 1,532 | 1,249 | Stratford and East-End District | 21,602 | 340,024 | 212,737 | 89,299 | 62,770 | 4,258 | 156,327 | 17,527 |
| 9,822 | 7,622 | | 104,202 | 1,621,449 | 1,061,662 | 452,194 | 276,941 | 73,157 | 802,292 | 265,778 |

### NEEDS OF EXTRA METROPOLITAN INSTITUTIONS.

NONE of the following institutions will benefit by the Hospital Sunday collections, but the managers desire to bring their claims before those who will see this supplement, by publishing their own statements on the page referred to opposite their names below, and we have pleasure in recording this fact here. This supplement is, we know, preserved by a large number of generous people for purposes of reference, and has for years constituted a handy guide to metropolitan hospitals and kindred institutions. No doubt this fact is becoming generally known throughout the country. We welcome it as an evidence that continuous effort in a good cause makes its results felt for good upon institutions and work done in districts far removed from the particular locality on behalf of the institutions of which it may have been directed.

**Association for Oral Instruction of Deaf and Dumb.**—£3,000 wanted to pay debt to bankers and to build a workshop (see p. 30).

**Bethnal Green Free Library.**—£300 in debt. Annual deficiency £2,000 (see p. 30).

**Birmingham Ear and Throat Hospital.**—Requires £2,500 to meet annual income (see p. 30).

**British Orphan Asylum, Slough.**—Deficit for 1901, £3,607; about £7,000 has to be raised from voluntary sources annually (see p. 29).

**Christian Community.**—Requires £3,000 annually (see p. 30).

**Infant Orphan Asylum, Wanstead.**—£2,500 wanted for drainage improvements (see p. 30).

**London Orphan Asylum, Watford.**—Requires £13,400 from voluntary sources to meet ordinary expenses (see p. 30).

**Rosehill Children's Hospital, Babbacombe, Torquay.**—£1,000 wanted for new building (see p. 29).

**National Refuges ("Arethusa" and "Chichester" Training Ships).**—Beyond reliable income, £10,000 is required for these charities (see p. 23).

**Wolverhampton Royal Orphanage.**—Expenditure exceeds income by £3,000 annually. Room for 100 more children but insufficient support (see p. 31).

## Figures and Tables

**Figure f1:**
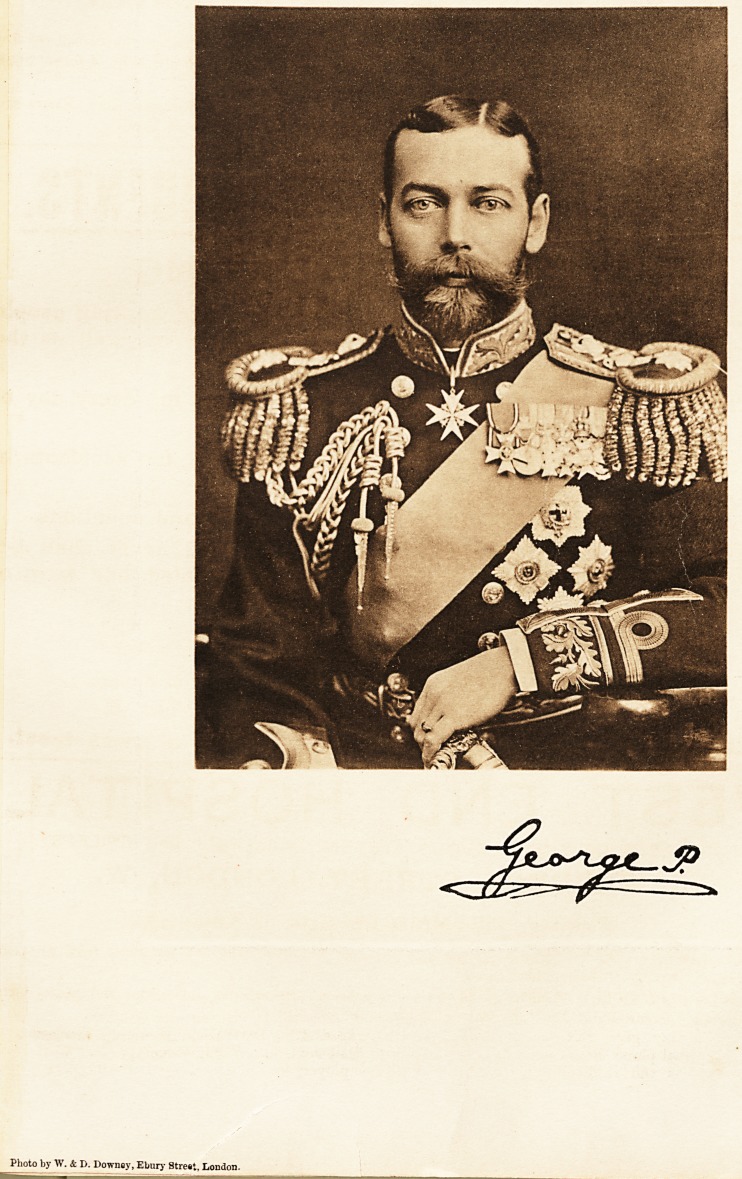


**Figure f2:**
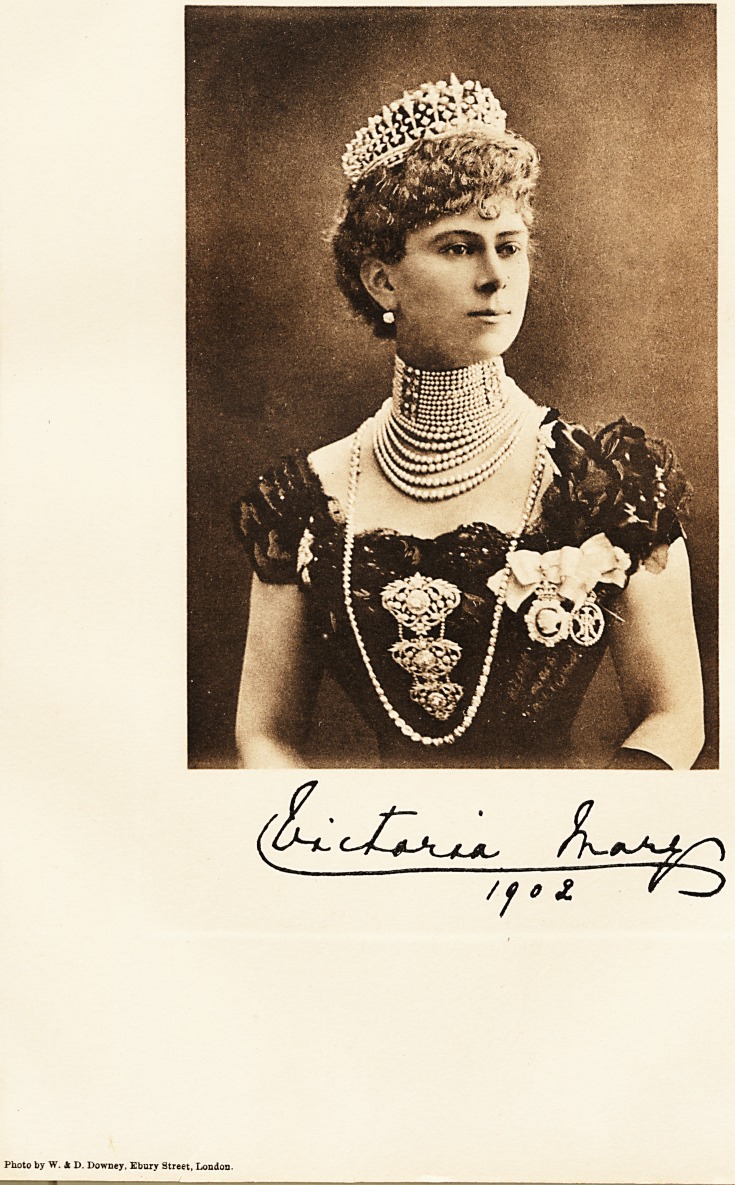


**Figure f3:**
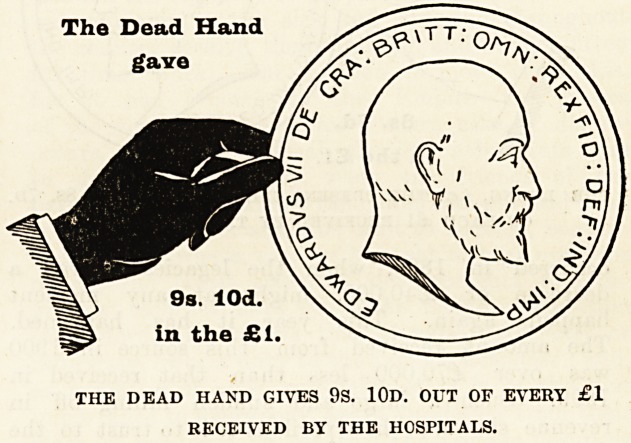


**Figure f4:**
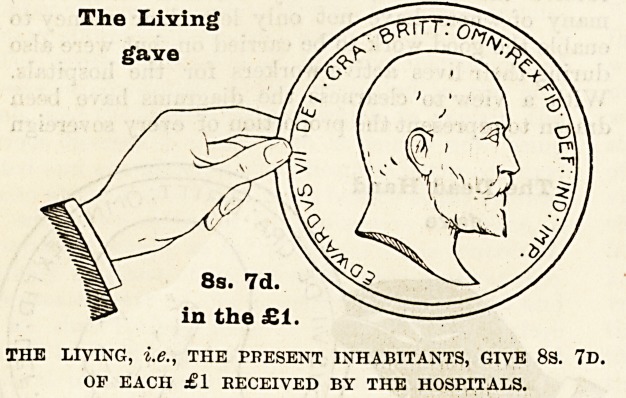


**Figure f5:**
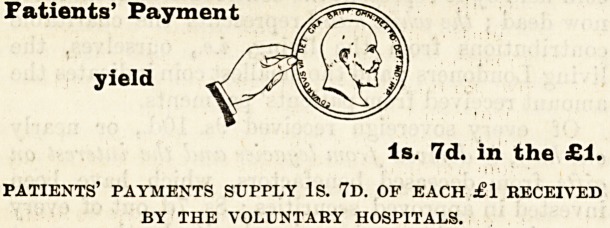


**Figure f6:**
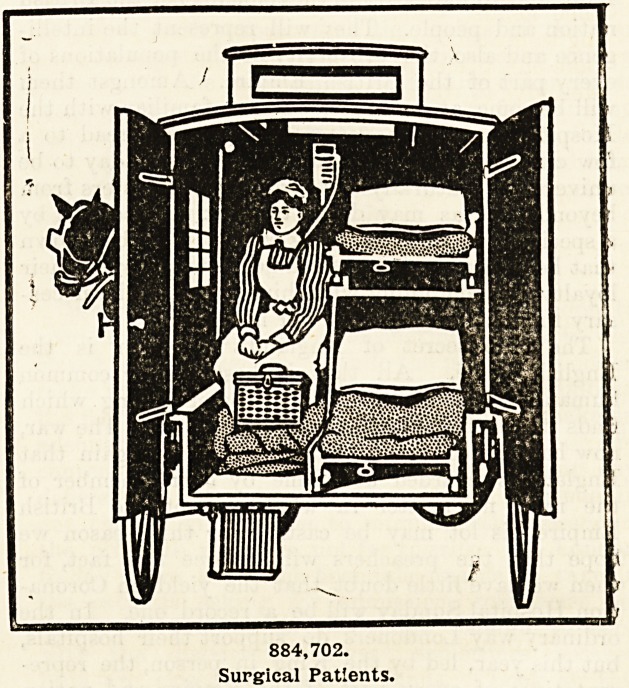


**Figure f7:**
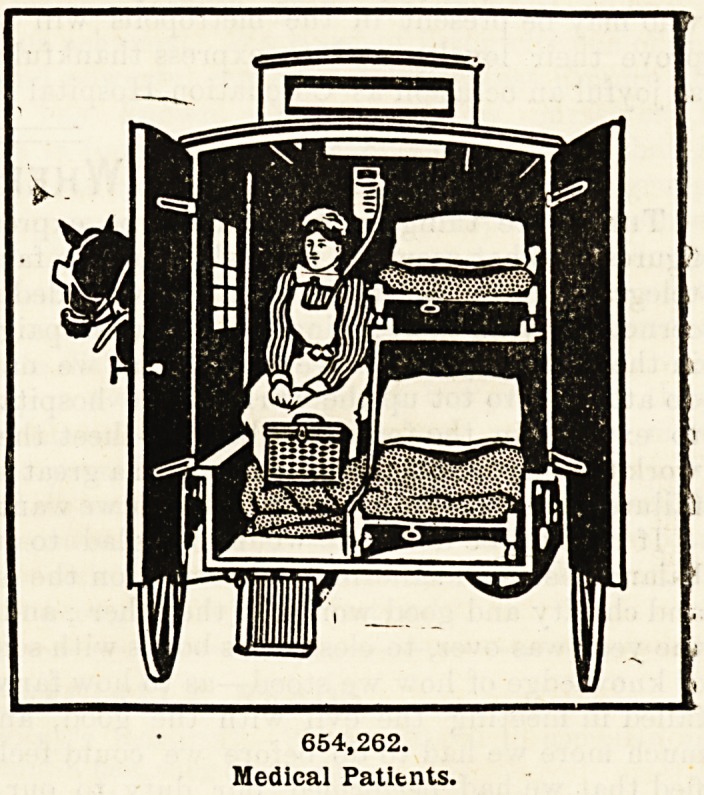


**Figure f8:**
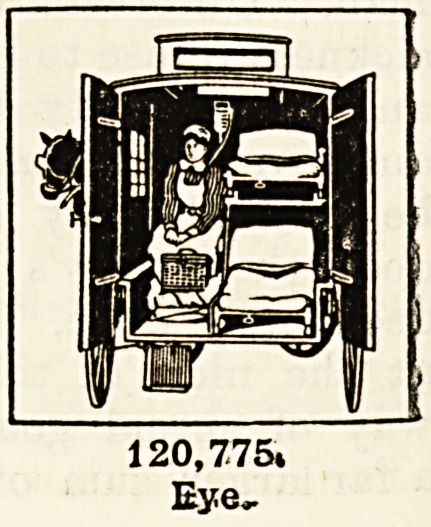


**Figure f9:**
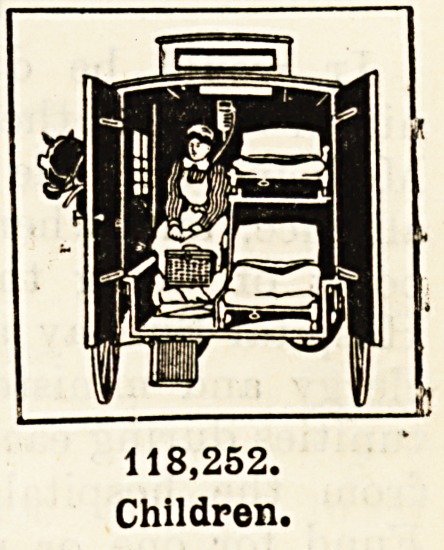


**Figure f10:**
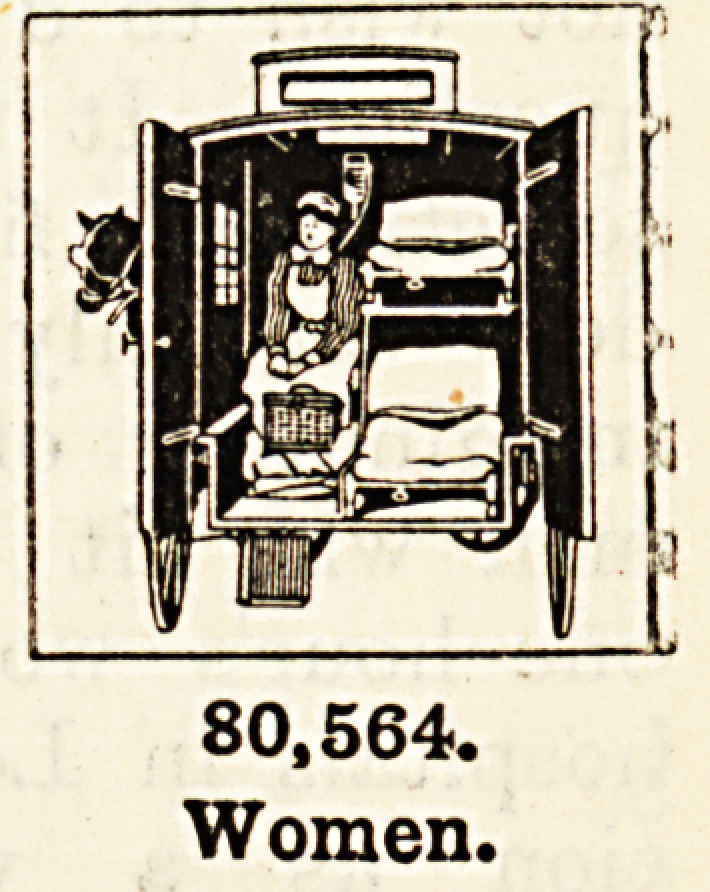


**Figure f11:**
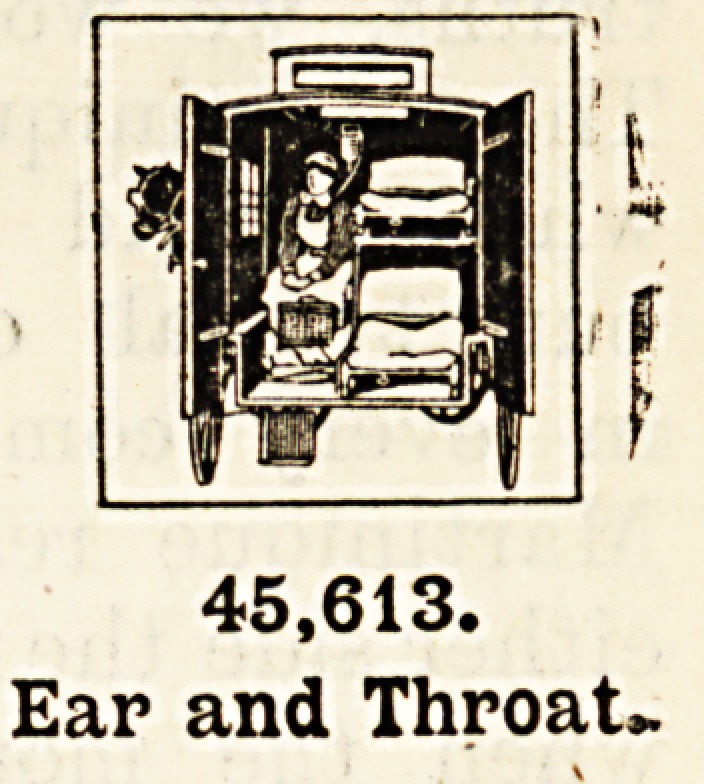


**Figure f12:**
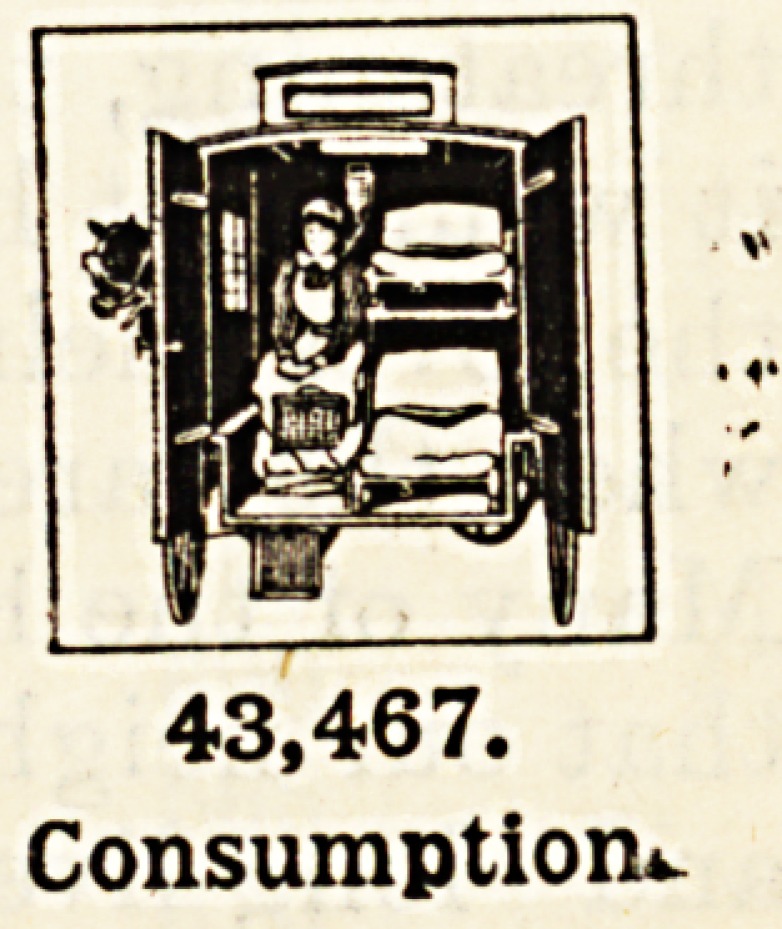


**Figure f13:**
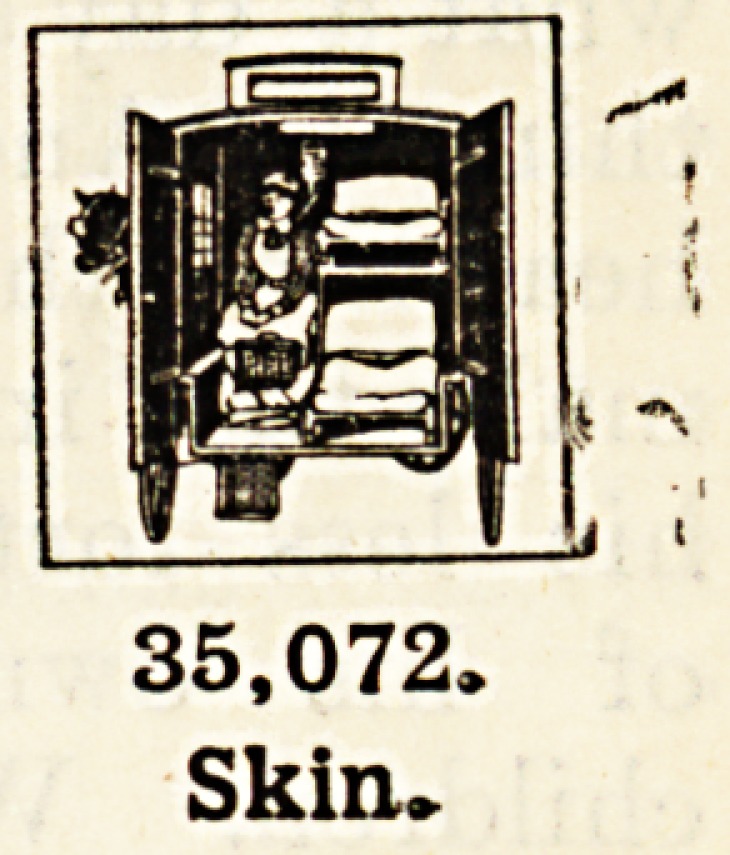


**Figure f14:**
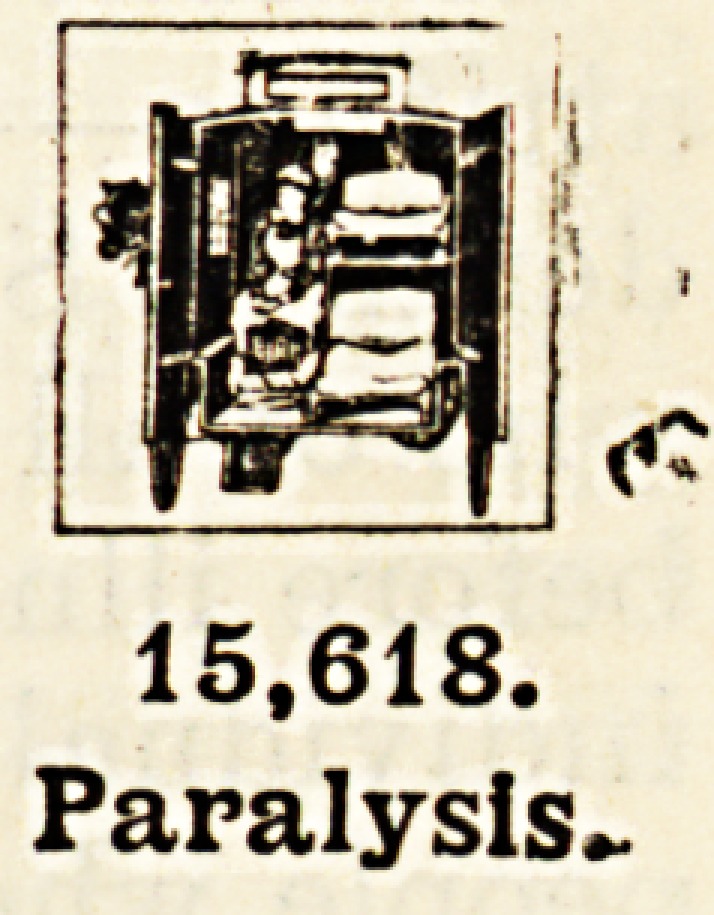


**Figure f15:**